# Sporadic Detection of *Escherichia coli* O104:H4 Strain C227/11Φcu in the Edible Parts of Lamb’s Lettuce Cultured in Contaminated Agricultural Soil Samples

**DOI:** 10.3390/microorganisms11082072

**Published:** 2023-08-12

**Authors:** Katharina Detert, Herbert Schmidt

**Affiliations:** Department of Food Microbiology and Hygiene, Institute of Food Science and Biotechnology, University of Hohenheim, Garbenstraße 28, 70599 Stuttgart, Germany; katharina.detert@uni-hohenheim.de

**Keywords:** EHEC/EAEC, *E. coli* O104:H4 C227/11Φcu, agricultural soil, edible part, plant colonization, lamb’s lettuce

## Abstract

In the current study, we demonstrate that *E. coli* O104:H4 strain C227/11Φcu, a derivative of the 2011 enterohemorrhagic/enteroaggregative (EHEC/EAEC) *E. coli* outbreak strain, migrated into the edible portion of lamb’s lettuce plants upon contamination of the surrounding soil. Seeds were surface-sterilized and cultivated on Murashige-Skoog agar or in autoclaved agricultural soil. Migration into the edible portions was investigated by inoculating the agar or soil close to the plants with 10^8^ colony-forming units (CFU). The edible parts, which did not come into contact with the contaminated medium or soil, were quantitatively analyzed for the presence of bacteria after 2, 4 and 8 weeks. Strain C227/11Φcu could colonize lamb’s lettuce when contamination of medium or soil occurs. The highest recovery rate (27%) was found for lettuce cultivated in agar, and up to 1.6 × 10^3^ CFU/g lettuce was detected. The recovery rate was lower for the soil samples (9% and 13.5%). Although the used contamination levels were high, migration of C227/11Φcu from the soil into the edible parts was demonstrated. This study further highlights the risk of crop plant contamination with pathogenic *E. coli* upon soil contamination.

## 1. Introduction

An increasing number of enterohemorrhagic *E. coli* (EHEC) outbreaks are correlated with consuming non-heated vegetables [1,2]. Between 2004 to 2012, 270 foodborne outbreaks occurred in the U.S., which were correlated with *E. coli* and 30 of these were linked to fresh produce, sprouts, leafy green, romaine lettuce or salad in general. The number increased from 2012 to 2021, and 341 foodborne *E. coli* outbreaks were detected. Of these, 50 outbreaks were attributed to the listed non-heated vegetables [3]. Based on recent and current outbreaks, the vegetable colonization ability of pathogenic *E. coli* O157:H7, which represents the dominant serotype correlated with foodborne outbreaks, has been investigated by several authors [4,5,6,7,8,9,10]. During the last years, romaine lettuce was a recurring contamination source for *E. coli* O157:H7 and mechanisms of plant contamination are not completely resolved [11,12]. Different studies demonstrated that EHEC can metabolize plant-derived nutrients, essential for colonization and survival in the plant environment [13,14]. In addition, EHEC was shown to tolerate biotic and abiotic stresses and thus, crop plants are assumed as secondary hosts for pathogenic *E. coli* [13,15,16,17]. Contamination of crop plants with EHEC can occur post-harvest or during the whole production chain of fresh produce [18]. Contaminations on the field can occur by wild animals or by using contaminated water and organic fertilizers [19,20,21,22,23]. By this, pathogenic bacteria can become in contact with plant surfaces and can enter the plant inside through stomata or other natural openings [24,25,26,27]. In addition, surface damage caused by plant pathogens and wounds or lesions can lead to leaf colonization by EHEC or other pathogens. One further contamination route of bacteria is the uptake by plant roots. In this case, the bacteria move and colonize inside the plants and cannot be removed through surface treatments such as washing. Since EHEC has a very low infectious dose, consuming non-heated contaminated vegetables can be a serious health risk for consumers.

In different studies, post-harvest contamination on the field and the colonization ability of human pathogens were analyzed [6,16,17,28]. Agricultural soil is an important contamination source, which has been underestimated. Human pathogens such as EHEC or *Salmonella enterica* can survive for several weeks in the soil [26,29,30,31,32]. Several studies analyzed the internalization capacity of pathogenic bacteria, resulting in many contradictory results [33]. Thereby, the inoculation process and the experimental setup are crucial. Different studies analyzed the internalization capacity of enteric pathogens into plant tissues, especially when plants were grown in inoculated hydroponic solution [34,35,36,37]. In contrast, only little or no internalization was found when plants were grown in contaminated soil [37,38]. Nevertheless, different studies showed that *E. coli* O157:H7 could colonize the roots and tissues of crop plants cultivated in contaminated soil [6,16,28,39].

Most studies addressing EHEC and crop plant contamination have focused on *E. coli* O157:H7, representing the most prevalent outbreak serogroup [40]. However, non-O157 but pathogenic *E. coli* correlated with serious outbreaks were less focused in such risk assessment studies. Consequently, *E. coli* O104:H4 strain C227/11 was chosen to represent a non-O157 EHEC strain associated with disease outbreaks after consumption of non-heated vegetables. The large EHEC/EAEC outbreak in Germany in 2011 was caused by the O104:H4 strain LB226692 [41]. *E. coli* O104:H4 strain C227/11 was then isolated from a German patient in Denmark [42]. The combination of virulence factors of the latter strain, which include the expression of Shiga toxin 2a (Stx2a) and the expression of aggregative adherence fimbriae, was correlated with the high pathogenic potential and the resulting severe courses of disease. Human infection was associated with raw sprout consumption, while the exact type of sprouts has still not been identified. Fenugreek sprouts were assumed to be the contamination source of this hybrid strain [2]. Since the pathogens were only rarely detected in foods and environmental samples at that time, it was hypothesized that the pathogens persist in the interior of the seeds or sprouts [43]. For laboratory safety reasons, this and former studies used the *stx2a*-negative derivative *E. coli* O104:H4 strain C227/11Φcu as an attenuated model strain [32,44].

A previous study showed that *E. coli* O104:H4 strain C227/11Φcu could internalize into the roots of lamb’s lettuce [44]. This motivated us to further analyze its colonization ability in lamb’s lettuce. If pathogenic bacteria colonize the interior of edible plants, they cannot be removed through surface treatment such as washing. Since EHEC strains have a low infectious dose of 10–100 cells [45], internal colonization of even low CFU numbers could result in a risk for consumers. This study focused on migration of C227/11Φcu into the plant through the root system to reach the edible portions of lamb’s lettuce using culture-dependent methods. The seeds were surface sterilized and cultivated in Murashige-Skoog agar (MS-agar) or autoclaved agricultural soil samples. When lamb’s lettuce plants reached the second leaf stage, the medium or soil was contaminated to mimic the incorporation of pathogenic bacteria on the field, e.g., via irrigation water or fertilizer. The edible parts of lamb’s lettuce were then analyzed for C227/11Φcu internalization and colonization.

## 2. Materials and Methods

### 2.1. Bacterial Strain

The *stx2a*-phage-cured derivative of *E. coli* O104:H4 strain C227/11 was used for the experiments [42]. This strain was chosen as a model strain with reduced virulence to allow safe laboratory work. *E. coli* O104:H4 strain C227/11Φcu was routinely grown in Luria-Bertani (LB) medium consisting of 10 g/L tryptone, 10 g/L NaCl and 5 g/L yeast extract (pH 7.0). Cultures were incubated in a rotary shaker at 37 °C and 180 rpm. For the preparation of solid agar plates, 15 g/L agar was added.

### 2.2. Seed Sterilization and Plant Cultivation

**Figure 1:**
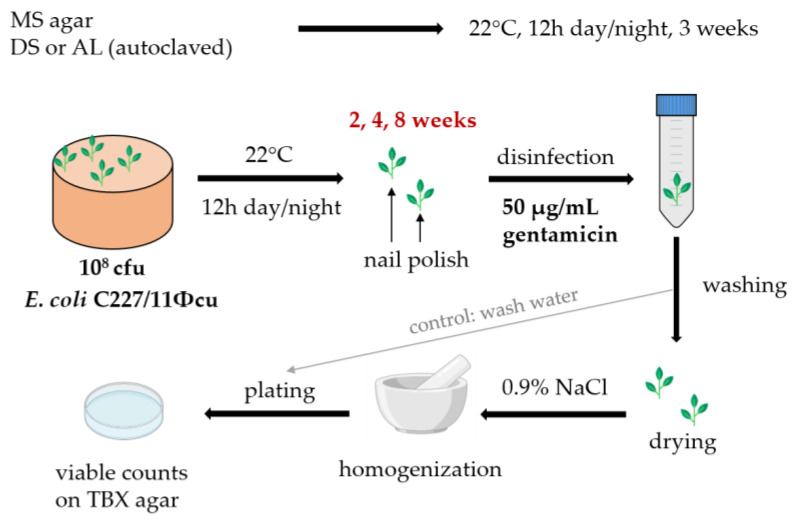
Experimental procedure for lamb’s lettuce cultivation, contamination with *E. coli* O104:H4 strain C227/11Φcu and sampling of edible parts.

The overall ability of *E. coli* O104:H4 strain C227/11Φcu to colonize the edible parts of lamb’s lettuce (*Valerianella locusta*) was investigated. Seeds of lamb’s lettuce were surface-sterilized in 50 µg/mL gentamicin for 20 min at room temperature. Afterwards, the seeds were washed three times with sterile demineralized water, dried on sterile filter paper and stored in the dark at room temperature until cultivation. Lamb’s lettuce seeds were cultivated either in 50 g agricultural soil samples or in 50 mL 0.5 × Murashige-Skoog (MS) agar (2.165 g/L Murashige & Skoog Medium, Duchefa Biochemie, Haarlem, Netherlands, pH 5.8). For this, 50 g Alluvial loam (AL) or Diluvial sand (DS), which were used in previous studies [26,32,39,44,46,47], were autoclaved at 121 °C for 15 min. Afterwards, the soil samples were transferred to plastic cups with diameters of 95 mm (Weber Packaging, Güglingen, Germany), and the soil was adjusted to 50% of its maximum water holding capacity (WHC_max_) using 10 mM MgCl_2_, as described earlier [26,46]. To each cup, four sterilized lamb’s lettuce seeds were transferred to the soil or the MS-agar at equal distances and cultivated for three weeks in a 12 h day/night cycle. After this, the plants reached the second leaf stage (first leaf rosette) and were inoculated. The further experimental procedure is shown graphically in Figure 1 and described below.

### 2.3. Plant Inoculation

For inoculation of the soil or agar around the lamb’s lettuce plants, overnight cultures of strain C227/11Φcu were used. The cells were harvested by centrifugation at 5000× *g* for 5 min and resuspended in the same volume of sterile 10 mM MgCl_2_. The OD_600_ of this suspension was measured, and the soil or the agar around the plants was spot-inoculated with 10^8^ colony-forming units (CFU). As controls, soil or agar was inoculated with 10 µL of 10 mM MgCl_2_. Inoculation was performed carefully to exclude direct contact with the roots/plants. The plants were then further incubated under the same conditions.

### 2.4. Plant Harvest

After 2, 4 and 8 weeks, the edible parts of lamb’s lettuce were sampled, which included all parts above the ground (stem and leaves) that were not in contact with the soil. These parts were cut off using a sterile scalpel, and the cutting sides were closed using commercial nail polish to prevent the flushing of internalized bacteria. After drying, eight plants from two plastic cups were pooled into a single 50 mL reaction tube (falcon) and surface-sterilized by dipping it in a 50 µg/mL gentamicin solution for 20 min at room temperature. After washing the sample three times with sterile demineralized water, the plants were dried on sterile filter paper and weighed. One sample consisted of three to four plants. After drying, the plants were transferred to a homogenization bag with a lateral filter (BBAG-03), and a 0.9% NaCl-solution was added to obtain a 1:10 dilution. The plants were homogenized using a mortar and incubated for 1 h at 22 °C.

After that, 100 µL of the mixture was plated directly on TBX agar, preparing four technical replicates. In addition, 1 mL of the homogenized solution was added to 9 mL of buffered peptone water. Enrichment was conducted for 24 h at 37 °C, and 100 µL of the samples were again plated directly on TBX agar. As a control, samples of the washing water were spread-plated to demonstrate that detected bacteria were not on the plant surface. The washing water was also enriched in buffered peptone water, as described above. The experiment was performed for each cultivation medium with four biological and two technical replicates resulting in 22 samples used for evaluation. Recovery rates were calculated as the percentage of the C227/11Φcu positive lamb’s lettuce samples with regard to all analyzed samples.

## 3. Results and Discussion

Crop plants may function as secondary hosts of enteric bacteria such as *Salmonella enterica* or EHEC [8,13,48]. Various studies focused on the interaction of bacterial pathogens and crop plants. Since crop plants are further processed into edible plants, mainly consumed raw, contaminations pose a health risk for consumers. In this study, the contamination of crop plants, which can occur on the field via irrigation water or organic fertilizer application, is analyzed in a model system. Agricultural soils are assumed to be an essential contamination source of plants. Successful colonization of the edible part of plants depends on bacterial survival in agricultural soils and adherence to root tissues. In addition, adapting to the nutrients plants provide is crucial for internal plant persistence. In previous studies, the survival of EHEC in the soil, as well as the genetic response during growth in lettuce medium, was investigated [14,32]. Different studies showed that *E. coli* survives for several weeks in the soil and that the reduction of soil microbiota by autoclaving even enhanced the survival period [30,32,49,50]. Bufe et al. (2019) [14] show that genes for chemotaxis and motility of different EHEC strains are upregulated when the bacteria are exposed to an artificial lettuce medium. *E. coli* O104:H4 strain C227/11Φcu can adapt and utilize the nutrients provided by the plants. Klerks et al. (2007) [51] further demonstrated that activation of bacterial chemotaxis by C-sources contained in root exudates led to bacterial movement towards the roots. In previous studies, the successful adherence and internalization into the roots of lettuce were also demonstrated for EHEC O157:H7 Sakai and O104:H4 C227/11Φcu [39,44].

To investigate whether C227/11Φcu can move into the edible parts of lamb’s lettuce via the vascular root system of the plants, we performed different experiments. Lamb’s lettuce was cultivated in MS-agar and the two different agricultural soil samples, AL and DS, as described above. We used sterilized lamb’s lettuce seeds and autoclaved the soil samples to reduce the number of competing soil bacteria. After the plants reached the second leaf stage, the agar or soil around the plants was inoculated with *E. coli* O104:H4 C227/11Φcu carefully, without touching the plants. The plants were further cultivated, and samples were taken after 2, 4 and 8 weeks of incubation. We analyzed the edible parts of lamb’s lettuce for the presence of C227/11Φcu. We analyzed the plants at different time points for each condition with two technical and four biological replicates summarized as four independent replicates. For this, up to four plants of one container were sampled and analyzed as one replicate. For each experimental set-up, 22 samples were analyzed for internalized bacteria using culture-dependent methods. The presence of O104:H4 strain C227/11Φcu was determined by CFU counts. In parallel, a non-specific enrichment was performed to decide whether the samples were positive but contained only CFU counts below the detection limit (<10 CFU/g). The ability to migrate into the edible parts via the root system of lamb’s lettuce was investigated, and the results are demonstrated in the following section. To verify that the phyllosphere of the plants was not inoculated accidentally, the washing water after plant surface disinfection was plated. In addition, the plants were inoculated only with 10 mM MgCl_2_. All controls showed no bacterial growth on the respective TBX agar plates. The ability to migrate into the edible parts via the root system of lamb’s lettuce was investigated, and the results are summarized in Table 1.

The results demonstrated in Table 1 show that *E. coli* O104:H4 strain C227/11Φcu can sporadically move into the edible portions of lamb’s lettuce following plant cultivation in contaminated agar or soil. Bacterial colonies were detected after 2 and 4 weeks when the plants were grown in MS agar (Table 1). Two of the four tested replicates were C227/11Φcu-positive at both time points, while two did not show internalized bacteria in the edible portions. In the positive samples, we detected up to 3.6 × 10^3^ CFU/g lamb’s lettuce after two weeks, while the numbers decreased for the samples taken after four weeks (Table 1). While one sample showed CFU counts close to the detection limit of 10 CFU/g, the second positive sample contained up to 10^2^ CFU/g. After eight weeks, we did not detect C227/11Φcu-positive samples. The non-selective enrichment of these samples did not result in bacteria detection indicating that the negative lettuce samples were not colonized. In this approach, we used more or less sterile conditions since surface-sterilized seeds and autoclaved MS-agar was used. By this, we achieved the highest recovery rate of 27%. Here, we detected C227/11Φcu in 6 out of 22 samples (Table 2).

For lamb’s lettuce plants cultivated in contaminated autoclaved AL, C227/11Φcu-positive samples were not found two weeks post-inoculation (Table 1). Enrichment of the samples also indicated that the plants were not colonized with C227/11Φcu. Two replicates were positive after four weeks, and one positive replicate was detected eight weeks post-inoculation (Table 1). After four weeks, only single colonies were detected on the agar plates resulting in CFU numbers close to the detection limit. After eight weeks, one replicate showed colonized edible portions and 6.9 × 10^3^ CFU/g lettuce was detected. The positive samples were further verified by unselective enrichment to prove that no *E. coli* other than those used for contamination was present. Indeed, in all negative samples, no C227/11Φcu bacteria growth was detected.

In summary, the cultivation of lamb’s lettuce plants in contaminated AL resulted in the detection of three C227/11Φcu-positive samples and thus in a recovery rate of 13.5% (Table 2). The lowest recovery rate of 9% was calculated for lamb’s lettuce plants, which were grown in contaminated DS (Table 2). C227/11Φcu was detected in one sample after two weeks of incubation with very low CFU numbers (Table 1). After four weeks, no further bacteria were detected in the edible parts of the tested plants. The highest CFU numbers of 2.0 × 10^3^ CFU/g lettuce were detected after eight weeks. In one of the four replicates, C227/11Φcu was detected in the edible portion of lamb’s lettuce.

The results demonstrated in Table 1 show that C227/11Φcu was detected in the edible portions. The CFU numbers were often close to the detection limit, and only low levels were detected. In some samples, higher CFU numbers up to 10^3^ CFU/g salad were detected. The study highlights that internalization occurs sporadically, which results in variability between the corresponding replicates. These findings are consistent with the results of other studies. As reviewed by Hirneisen et al. [33], different studies focused on the internalization of pathogenic *E. coli* into crop plants. For those studies that detected internalization in plants grown in contaminated soil, internalization occurred sporadically and at low levels, as shown in the present study [15,37,52,53].

Previous studies compared the internalization ability of pathogenic bacteria when plants were grown in autoclaved or non-autoclaved soils [37,54]. The study of Cooley et al. (2003) [54] demonstrated that the endogenous soil microbiota out-competed enteric bacteria such as *Salmonella* Newport or *E. coli* O157:H7 strains. Soils are complex mixtures of organic matter, minerals, water, gases, and numerous organisms, including spore-forming bacteria. In the current study, we autoclaved the soil samples before plant cultivation to reduce the amounts of competing bacteria. Here, we demonstrated that migration of C227/11Φcu in the edible portions occurred when lamb’s lettuce was cultivated in autoclaved soil samples. The highest recovery rate was found for C227/11Φcu when plants were grown in sterile MS-agar. This suggests that the soil’s biotic and abiotic factors might diminish bacterial migration into the edible plant parts. This assumption is supported by others [33]. In addition, factors such as plant type, pathogen strain and inoculum level must be considered. In the current study, we used high inoculation levels of 10^8^ CFU/mL, which are unusual for overall natural field contamination levels. Nevertheless, CFU numbers up to 10^8^ CFU/g soil can be found locally on the field, especially after fecal contamination. The same inoculation levels were used in the study of Eißenberger et al. (2020) [44], where the root adherence and internalization of C227/11Φcu were investigated. The authors also used DS and AL for plant cultivation and detected 2.6 × 10^6^ CFU/g root and 8.7 × 10^5^ CFU/g root at the roots of lamb’s lettuce. Compared to that, the number of internalized bacteria was reduced. The authors detected 4.1 × 10^2^ CFU/g root to 2.4 × 10^2^ CFU/g root of strain C227/11Φcu in the roots of lamb’s lettuce after growth in DS and AL, respectively. In the present study, we observed the movement of strain O104:H4 C227/11Φcu into the edible parts of lamb’s lettuce, indicating that the bacteria could internalize into the roots under the tested conditions. C227/11Φcu-positive plants showed CFU numbers of 1.3 × 10^1^ to 6.9 × 10^3^ CFU/g lettuce (Table 1). These numbers are in the range of internalized bacteria found by Eißenberger et al. (2020) [44], which led us to hypothesize that internalized bacteria further spread within the lettuce plant and colonize the edible portions of the plant. It is assumed that bacteria might move and colonize the plants by using the vascular system of the plants. Jechalke et al. (2019) [26] identified secondary root emerging zones and root hairs as potential internalization routes of *Salmonella* Typhimurium and demonstrated that the former one is preferentially colonized by bacteria. The authors assumed this zone was nutrient-rich and attractive for bacterial colonization as it is also known for the rhizosphere. *E. coli* accumulate mostly on or in the roots of plants and in rhizosphere soil. The number of adhered bacteria compared to internalized bacteria was significantly higher in the study of Eißenberger et al. (2020) [44]. The data presented in this study further highlighted that more bacteria adhere to the roots than internalize into the roots of lamb’s lettuce. The low internalization rate might be the reason for the low number of positive colonization samples and the resulting recovery rates (Table 2). In the study of Jechalke et al. (2019) [26], *Salmonella enterica* was detected in 0.3–0.4% of the lamb’s lettuce samples. In a further study, *Salmonella* was detected in 2.9% of the surface sterilized lettuce plants corresponding to one positive sample out of 35 [55]. Compared to that, we received higher recovery rates of 9–27% and at least two and up to six positive samples out of 22.

Different studies demonstrated that the frequency of plant colonization by EHEC O157:H7 is largely influenced by the plant type [8,17]. Chitarra et al. (2014) [17] found no internalization of *E. coli* O157:H7 in basil that might correlate with the antibacterial activity of essential oils contained in basil. For broccoli, lettuce and rocket, sporadic colonization of EHEC O157:H7 Sakai of about 15% was detected. In comparison, plants with colonized leaves were detected for 50% parsley plants and more than 75% alfalfa and coriander plants [8]. The study further highlighted that *E. coli* O157:H7 was isolated from cotyledons of all tested plant species when contamination occurred during the germination of seeds. Lamb’s lettuce is consumed, including the cotyledons, a few weeks after germination. If the seeds are exposed to contaminated soil or water, colonization of the plants is possible.

## 4. Conclusions

The current study confirmed the successful migration of the pathogenic *E. coli* strain C227/11Φcu into the edible portions of lamb’s lettuce when the plants were grown in contaminated soil. The detectable amounts of C227/11Φcu were low, and internalization occurred sporadically. Given the inoculation of the soil samples with high numbers of 10^8^ CFU, approx. 10^3^ CFU/g lettuce were detected after eight weeks, estimating approximately a reduction of5 log. Therefore, a risk of human infection is only expected when fertilization with highly contaminated manure is carried out. However, exposure of lamb’s lettuce to contaminated soil or irrigation water represents an ongoing food safety risk and should be analyzed on a larger scale, e.g., in greenhouses.

## Figures and Tables

**Table 1:** *E. coli* O104:H4 C227/11Φcu was detected in surface-disinfected edible portions of lamb’s lettuce cultivated in MS agar, AL or DS at different time points.

	Time Point	C227/11Φcu-Positive Samples * (*n*)	CFU/g Lamb’s Lettuce **	
			**Sample 1**	**Sample 2**
MS agar	2 weeks	2 (4)	3.6 × 10^3^	1.1 × 10^3^
	4 weeks	2 (4)	2.5 × 10^1^	1.5 × 10^2^
	8 weeks	0 (4)	n.d.	n.d.
Alluvial loam (AL)	2 weeks	0 (4)	n.d.	n.d.
	4 weeks	2 (4)	1.3 × 10^1^	1.3 × 10^1^
	8 weeks	1 (4)	6.9 × 10^3^	n.d.
Diluvial sand (DS)	2 weeks	1 (4)	2.5 × 10^1^	n.d.
	4 weeks	0 (4)	n.d.	n.d.
	8 weeks	1 (4)	2.0 × 10^3^	n.d.

n.d. = not detected. * number of samples containing internalized C227/11Φcu and the total number (*n*) of analyzed lamb’s lettuce plants (the experiments were repeated in four independent replicates). ** Viable counts of internalized bacteria shown for the C227/11Φcu-positive replicates detected at the respective time points given in CFU/g lamb’s lettuce.

**Table 2:** C227/11Φcu -positive lamb’s lettuce samples with the calculated recovery rate.

Soil Type/Agar	Samples	Positive Samples	Recovery Rate
MS	22	6	27%
AL	22	3	13.5%
DS	22	2	9%

## Data Availability

The datasets generated and/or analyzed during the current study are available from the corresponding author upon reasonable request.
